# Preventive and Interceptive Orthodontic Treatment Needs in Early and Late Mixed Dentition: An IPION-Based Assessment in Children

**DOI:** 10.3390/children13091252

**Published:** 2026-09-15

**Authors:** Nuri Can Tanrısever, Tuğba Bezgin, Tülin Ufuk Toygar Memikoğlu

**Affiliations:** 1 Department of Orthodontics, School of Dentistry, Ankara University, 06560 Ankara, Türkiye; 2 Department of Pediatric Dentistry, School of Dentistry, Ankara University, 06560 Ankara, Türkiye

**Keywords:** child, dentition, mixed, index of preventive and interceptive orthodontic needs (IPION), malocclusion, orthodontics, interceptive, orthodontics, preventive

## Abstract

**Highlights:**

**What are the main findings?**

- Preventive/interceptive orthodontic treatment was common in both early and late mixed dentition, with definite treatment need being the most frequent category in both age groups.
- Dental caries and premature loss of primary teeth were more strongly related to the overall IPION profile during early mixed dentition.

**What are the implications of the main findings?**

- Age-specific IPION assessment may help identify children who may benefit from further orthodontic evaluation during mixed dentition.
- Integrating dental, developmental, and occlusal findings may support earlier referral and closer collaboration between pediatric dentists and orthodontists.

**Abstract:**

**Background/Objectives**: Mixed dentition is critical for identifying developing malocclusions that may benefit from preventive or interceptive orthodontic management. This study aimed to assess IPION-defined preventive/interceptive orthodontic treatment need using the Index of Preventive and Interceptive Orthodontic Needs (IPION), compare early and late mixed dentition, and evaluate associated clinical findings. **Methods**: This single-center cross-sectional study included 200 children attending their first examination at the university Pediatric Dentistry and/or Orthodontics clinics: 100 aged 6 and 100 aged 9 years. IPION-defined treatment need was assessed using the age-specific IPION-6 and IPION-9 subsystems. Dental, developmental, and occlusal findings were recorded using standardized criteria. Treatment-need categories and clinical findings common to both subsystems were compared between groups, while total IPION scores were summarized separately by age. Relationships of caries and premature primary tooth loss with IPION scores were evaluated. **Results**: Definite treatment need predominated (58.0% vs. 51.0%), and treatment-need distributions differed between age groups (*p* = 0.047). Dental caries, molar relationship, overjet, and overbite showed significant differences. In 6-year-olds, carious and prematurely lost tooth counts were positively correlated with IPION scores, with the strongest relationship for premature tooth loss (ρ = 0.609; *p* < 0.001). Relationships were weaker in 9-year-olds. **Conclusions**: IPION-defined preventive/interceptive orthodontic treatment need was common within this university-based clinical sample across mixed dentition stages. Differences in treatment-need distribution and clinical findings indicated distinct preventive and interceptive orthodontic profiles across mixed dentition stages. Age-specific IPION assessment may support identification of children who may benefit from further orthodontic evaluation by integrating dental, developmental, and occlusal findings.

## 1. Introduction

The mixed dentition period represents a critical stage of craniofacial growth and dental development, during which many developing malocclusions first become clinically detectable [1,2]. Timely recognition of these conditions can help limit their progression into more complex orthodontic problems requiring comprehensive treatment in the permanent dentition [3,4], particularly in cases involving transverse discrepancies, developing space deficiencies, functional shifts, and other occlusal disturbances that can adversely affect subsequent dentofacial development [5,6,7]. Preventive and interceptive orthodontics aim to utilize the growth potential of the developing dentition by eliminating etiological factors, preventing the establishment of malocclusion, or intercepting developing occlusal problems before they become more severe [8]. In appropriately selected cases, early orthodontic intervention can reduce treatment complexity, facilitate more favorable dentofacial development, and potentially shorten or simplify subsequent comprehensive treatment [9]. Accordingly, the early identification of children who may benefit from preventive or interceptive orthodontic management represents an important component of pediatric dental care and also supports the planning and allocation of oral health services [10,11,12].

Several indices, including the Dental Aesthetic Index (DAI), the Index of Orthodontic Treatment Need (IOTN), and the Index of Complexity, Outcome and Need (ICON), have been developed to provide standardized assessments of orthodontic treatment need [13,14,15]. However, these indices were designed primarily for the permanent dentition and have limited applicability during the mixed dentition period because they do not fully account for age-specific developmental characteristics and clinical conditions encountered during this transitional stage [16]. These include ongoing exfoliation and eruption, premature loss of primary teeth, caries-related space changes, and changes in arch space associated with the transition from primary to permanent successors, including the utilization of leeway space. Such features can influence the developing occlusion before a definitive permanent-dentition malocclusion is established. Consequently, the assessment of orthodontic treatment need in children requires approaches that consider not only established malocclusions but also developmental conditions with the potential to influence subsequent occlusal development.

To address this limitation, the Index of Preventive and Interceptive Orthodontic Needs (IPION) was specifically developed for children in the mixed dentition period [17]. The index comprises two age-specific subsystems, IPION-6 and IPION-9, which use weighted clinical findings and age-specific thresholds to classify preventive and interceptive orthodontic treatment need. In addition to established occlusal abnormalities, the IPION incorporates clinical conditions such as dental caries and premature tooth loss that can influence dental arch development and subsequent orthodontic treatment need. By integrating these developmental and clinical factors, the IPION provides an epidemiological screening approach for identifying children who may benefit from early orthodontic assessment and management [16,17,18,19,20].

Previous studies have used the IPION to estimate the prevalence of preventive and interceptive orthodontic treatment need in different populations [16,18,19]. However, evidence regarding the distribution of the individual clinical findings contributing to IPION scores across different stages of mixed dentition remains limited. Examining these findings separately in the early and late mixed dentition periods provides a more detailed understanding of how preventive and interceptive orthodontic needs vary throughout dental development. Such information helps identify the clinical conditions that predominate at different developmental stages and can inform age-appropriate screening, referral, and early orthodontic management. Furthermore, population-specific data remain important because the prevalence and distribution of these findings may vary according to demographic, environmental, and oral health-related characteristics.

Therefore, this study aimed to assess IPION-defined preventive and interceptive orthodontic treatment need and to compare treatment-need categories and the distribution of associated clinical findings between children in the early and late mixed dentition periods. The null hypothesis was that there would be no differences between the two age groups in the distribution of IPION-defined treatment-need categories or in clinical findings common to both age-specific subsystems.

## 2. Materials and Methods

### 2.1. Study Design and Participants

This cross-sectional observational study was conducted at the Departments of Orthodontics and Pediatric Dentistry, Faculty of Dentistry, Ankara University, Ankara, Türkiye. The study protocol was approved by the Non-Interventional Clinical Research Ethics Committee of Ankara University Faculty of Dentistry (Meeting No. 14; approval date: 6 October 2025). The study was conducted in accordance with the principles of the Declaration of Helsinki. Written informed consent was obtained from the legal guardians of all participants, and assent was obtained from the children prior to enrollment.

Children presenting for their first clinical examination at the Departments of Pediatric Dentistry and/or Orthodontics were screened for eligibility during the study period, and those meeting the predefined criteria were included until 100 participants were enrolled in each age group. A total of 200 children in the mixed dentition period were included: 100 children aged 6 years, representing the early mixed dentition period, and 100 children aged 9 years, representing the late mixed dentition period.

Children in the mixed dentition period who had not previously undergone orthodontic treatment and were able to cooperate during the clinical examination were eligible for inclusion. Children with craniofacial syndromes, cleft lip and/or palate, systemic diseases potentially affecting craniofacial growth and development, or a history of previous orthodontic treatment were excluded.

### 2.2. Clinical Examination and IPION Assessment

Clinical examinations were performed by two experienced specialists from the Departments of Pediatric Dentistry and Orthodontics according to the standardized assessment protocol of the Index of Preventive and Interceptive Orthodontic Needs (IPION). Before data collection, the examiners jointly reviewed the operational definitions, measurement procedures, scoring thresholds, and classification criteria for each IPION parameter to standardize the clinical assessment process and minimize examiner-related variability. All examinations were performed using the same predefined clinical criteria and measurement procedures, with a dental mirror, explorer, and millimetric ruler under clinical conditions.

Participants were evaluated using the age-specific IPION subsystem corresponding to their chronological age: IPION-6 for children aged 6 years and IPION-9 for children aged 9 years. The two subsystems are structured according to the developmental characteristics of their respective mixed-dentition stages. Accordingly, IPION-9 includes additional dental and developmental findings relevant to the later mixed-dentition stage, including supernumerary teeth, eruption disturbances, active frenulum, missing permanent incisors, and midline diastema. The clinical parameters and scoring criteria included in the IPION assessment are presented in Table 1.

All clinical parameters were assessed by direct intraoral examination. Overjet and midline diastema were measured in millimeters using a millimetric ruler, whereas overbite was evaluated according to the extent of vertical overlap of the maxillary and mandibular incisors. Sagittal molar relationships and anterior and posterior crossbites were evaluated in maximum intercuspation. When the permanent first molars had not fully erupted, or their relationship could not be reliably determined, the relationship of the primary second molars was used as the reference for sagittal occlusal assessment.

Dental caries and premature loss of primary canines and molars (III, IV, and V) were assessed exclusively by visual–tactile clinical examination by visual–tactile clinical examination. Lip incompetence was assessed according to the resting lip posture. Supernumerary teeth, eruption disturbances or impaction of the primary first and second molars (IV and V), active frenulum, missing permanent incisors, and midline diastema were assessed only within the IPION-9 subsystem.

### 2.3. IPION Scoring and Classification of Treatment Need

For each participant, a total IPION score was calculated according to the original scoring system described by Coetzee et al. [17]. The age-specific thresholds recommended for the IPION-6 and IPION-9 subsystems were used to classify IPION-defined preventive and interceptive orthodontic treatment need. Because the two subsystems differ in their item composition and scoring structure, treatment-need classification was based on the specific thresholds defined for each age-specific subsystem.

For IPION-6, treatment need was categorized as follows:

- 0–4: No treatment needed;
- 5–13: Moderate treatment needed;
- ≥14: Definite interceptive treatment needed.

For IPION-9, treatment need was categorized as follows:

- 0–5: No treatment needed;
- 6–14: Moderate treatment needed;
- ≥15: Definite interceptive treatment needed.

### 2.4. Outcome Measures

The principal study outcomes were the total IPION score and the corresponding IPION-defined preventive/interceptive orthodontic treatment-need category. Treatment-need categories were compared between the 6-year-old and 9-year-old groups, whereas total IPION scores were summarized separately within each age-specific subsystem.

In addition, clinical parameters common to both IPION-6 and IPION-9 were compared between age groups to characterize the pattern of preventive and interceptive orthodontic needs at different stages of mixed dentition. Findings assessed exclusively within the IPION-9 subsystem were summarized descriptively. The numbers of teeth affected by dental caries and premature tooth loss were also recorded separately at the participant level for subsequent association analyses.

### 2.5. Statistical Analysis

An a priori sample size calculation was performed using G*Power software (Version 3.1.9.7; Heinrich Heine University, Düsseldorf, Germany). The sample size calculation was based on the total IPION score, using an anticipated standardized effect size of d = 0.40 derived from previous IPION research [16]. With a significance level of α = 0.05, a statistical power of 80% (1 − β = 0.80), and an allocation ratio of 1:1, a minimum of 100 participants per group (200 participants in total) was required.

Statistical analyses were performed using IBM SPSS Statistics for Windows (Version 26.0; IBM Corp., Armonk, NY, USA). Continuous variables were summarized as mean ± standard deviation and median (minimum–maximum), whereas categorical variables were presented as frequencies and percentages.

The distribution of continuous variables was evaluated using histograms, Q–Q plots, and the Shapiro–Wilk test. As the continuous variables did not satisfy the assumption of normality, nonparametric statistical methods were used for inferential analyses.

Total IPION scores were summarized separately within each age-specific subsystem. Age-group comparisons were performed for treatment-need categories and clinical findings common to IPION-6 and IPION-9.

The distributions of preventive/interceptive orthodontic treatment-need categories and individual clinical findings were compared between age groups using Pearson’s chi-square test. When the assumptions of the chi-square test were not satisfied because of low expected cell frequencies, *p*-values were estimated using the Monte Carlo simulation method. The strength of associations between categorical variables was quantified using Cramér’s V.

Within each age group, associations between the number of carious teeth, the number of prematurely lost primary teeth, and total IPION score were assessed using Spearman’s rank correlation coefficient (*r*s). The numbers of carious teeth and prematurely lost primary teeth were also compared across treatment-need categories using the Kruskal–Wallis H test. When a statistically significant overall difference was detected, pairwise comparisons were subsequently performed using the Mann–Whitney U test with Bonferroni adjustment for multiple comparisons. When only two treatment-need categories were represented within an age group, the Mann–Whitney U test was used directly.

All statistical tests were two-tailed, and statistical significance was set at *p* < 0.05.

## 3. Results

A total of 200 children were included in the study, with 100 participants in each age group. The 6-year-old group comprised 53 girls and 47 boys, whereas the 9-year-old group comprised 41 girls and 59 boys.

### 3.1. IPION Scores and Treatment Need

The mean total IPION score was 15.20 ± 10.40 in the 6-year-old group and 19.30 ± 12.30 in the 9-year-old group, with median scores of 14 and 17, respectively.

According to the age-specific IPION classification, Definite treatment need was the most frequent category in both age groups, affecting 51.0% of the 6-year-old group and 58.0% of the 9-year-old group. Moderate treatment need was observed in 35.0% and 38.0% of the groups, respectively, whereas the proportion of children with no treatment need decreased from 14.0% in the 6-year-old group to 4.0% in the 9-year-old group. The overall distribution of treatment-need categories differed significantly between the age groups (χ^2^ = 6.12, *p* = 0.047) (Table 2).

### 3.2. Distribution of Dental and Occlusal Findings

The distribution of dental caries differed significantly between the age groups (χ^2^ = 20.30, *p* < 0.001; Cramér’s V = 0.319). Half of the children in the 6-year-old group were caries-free, compared with 32.0% of those in the 9-year-old group. Occlusal caries was more frequent in the 9-year-old group than in the 6-year-old group (57.0% vs. 26.0%), whereas interproximal caries was more frequent in the 6-year-old group (24.0% vs. 11.0%).

Premature tooth loss was present in 45.0% of the 6-year-old group and 57.0% of the 9-year-old group; however, the difference between the age groups was not statistically significant (χ^2^ = 2.80, *p* = 0.091; Cramér’s V = 0.118).

Permanent molar relationship differed significantly between the age groups (χ^2^ = 6.70, *p* = 0.035; Cramér’s V = 0.183). A Class I relationship was more frequent in the 6-year-old group, whereas Class II and Class III relationships were more frequent in the 9-year-old group.

The distribution of overjet also differed significantly between the age groups (χ^2^ = 16.10, Monte Carlo *p* = 0.001; Cramér’s V = 0.284). An overjet of 0–3 mm was observed in 82.0% of the 6-year-old group and 59.0% of the 9-year-old group, whereas increased overjet categories were more frequent in the older group.

A significant difference was also observed in the distribution of overbite (χ^2^ = 23.40, *p* < 0.001; Cramér’s V = 0.342). Coverage exceeding two-thirds of the mandibular incisor crowns and complete coverage were more frequent in the 9-year-old group. In contrast, the distribution of anterior open bite did not differ significantly between the groups (χ^2^ = 9.10, Monte Carlo *p* = 0.099; Cramér’s V = 0.213).

No statistically significant differences between the age groups were observed for anterior crossbite (χ^2^ = 2.80, *p* = 0.237; Cramér’s V = 0.118), posterior crossbite (χ^2^ = 8.40, Monte Carlo *p* = 0.078; Cramér’s V = 0.205), or lip incompetence (χ^2^ = 0.503, Monte Carlo *p* = 0.895; Cramér’s V = 0.050). Lip incompetence was absent in 83.0% of children in the 6-year-old group and 86.0% of those in the 9-year-old group (Table 3).

Among the additional clinical findings assessed exclusively within the IPION-9 subsystem, supernumerary teeth were identified in 8.0% of children, eruption disturbance or impaction involving at least one primary molar in 19.0%, and at least one missing permanent incisor in 13.0%. Active frenulum was present in 4.0% of children, while a midline diastema >2 mm was identified in 32 of the 93 assessable children (34.4%). These age-specific findings were summarized descriptively because they were not assessed in the IPION-6 group.

### 3.3. Associations of Caries and Premature Tooth Loss with Total IPION Scores

In the 6-year-old group, the number of carious teeth showed a moderate positive correlation with the total IPION score (Spearman’s ρ = 0.401, *p* < 0.001). In contrast, no statistically significant correlation was observed in the 9-year-old group (ρ = 0.154, *p* = 0.127).

The number of prematurely lost teeth was positively correlated with the total IPION score in both age groups. The correlation was strong in the 6-year-old group (ρ = 0.609, *p* < 0.001) and weak in the 9-year-old group (ρ = 0.216, *p* = 0.031) (Table 4).

### 3.4. Caries and Premature Tooth Loss According to Treatment Need

Among 6-year-olds, the number of carious teeth differed significantly across treatment-need categories (Kruskal–Wallis H = 15.43, *p* < 0.001). Descriptively, the mean number of carious teeth increased from 0.07 ± 0.27 in children with no treatment need to 0.74 ± 0.89 in the moderate-need group and 1.12 ± 0.91 in the definite-need group. Bonferroni-adjusted pairwise comparisons showed significant differences between all three treatment-need categories.

The number of prematurely lost teeth also differed significantly across treatment-need categories in the 6-year-old group (Kruskal–Wallis H = 23.70, *p* < 0.001). Descriptively, the mean number of prematurely lost teeth increased from 0.07 ± 0.27 in the no-treatment-need group to 0.43 ± 0.92 in the moderate-need group and 1.10 ± 1.01 in the definite-need group. Bonferroni-adjusted pairwise comparisons showed significant differences between all three categories.

In the 9-year-old group, the number of carious teeth did not differ significantly across treatment-need categories (Kruskal–Wallis H = 2.17, *p* = 0.339). However, the number of prematurely lost teeth differed significantly across the three treatment-need categories (Kruskal–Wallis H = 6.30, *p* = 0.043). Descriptively, the mean number of prematurely lost teeth increased from 0.00 ± 0.00 in the no-treatment-need group to 0.66 ± 0.78 in the moderate-need group and 0.90 ± 0.93 in the definite-need group. Nevertheless, none of the Bonferroni-adjusted pairwise comparisons reached statistical significance (Table 5).

### 3.5. Caries and Premature Tooth Loss According to Treatment-Need Categories

In the 6-year-old group, the distribution of caries categories differed significantly across treatment-need categories (χ^2^ = 15.01, Monte Carlo *p* = 0.003; Cramér’s V = 0.274). Definite treatment need was observed in 36.0% of caries-free children, 61.5% of those with occlusal caries, and 70.8% of those with interproximal caries.

Premature tooth loss status also differed significantly across treatment-need categories among 6-year-olds (χ^2^ = 24.80, *p* < 0.001; Cramér’s V = 0.498). Definite treatment need was observed in 77.8% of children with premature tooth loss compared with 29.1% of those without premature tooth loss.

In the 9-year-old group, neither caries category (χ^2^ = 1.50, Monte Carlo *p* = 0.881; Cramér’s V = 0.087) nor premature tooth loss status (χ^2^ = 5.50, Monte Carlo *p* = 0.055; Cramér’s V = 0.235) differed significantly across treatment-need categories (Table 6).

## 4. Discussion

To our knowledge, this study is among the first to evaluate IPION-defined preventive and interceptive orthodontic treatment need using the age-specific IPION system while simultaneously comparing the distribution of associated clinical findings between children in the early and late mixed dentition periods. Overall, moderate to definite IPION-defined treatment need was common within this university-based clinical sample, and the distribution of treatment-need categories differed between the two stages of mixed dentition. Although definite treatment need predominated in both groups, age-related differences were identified in several dental and occlusal findings, particularly dental caries, sagittal occlusal relationships, overjet, and overbite. Furthermore, dental caries and premature loss of primary teeth contributed more prominently to the overall IPION profile during the early mixed dentition period. Because the distribution of IPION-defined treatment-need categories and several clinical findings common to both age-specific subsystems differed significantly between the two age groups, the null hypothesis was rejected.

The IPION-6 and IPION-9 subsystems are designed for different stages of mixed dentition and therefore differ in the clinical characteristics included in their scoring structures. As children progress through the mixed dentition, occlusal discrepancies may become more apparent with the eruption of additional permanent teeth and the establishment of permanent molar relationships [1,9]. Moreover, unlike IPION-6, the IPION-9 subsystem incorporates additional age-specific variables, including eruption disturbances, supernumerary teeth, missing permanent incisors, active frenulum, and midline diastema [17]. These differences reflect the developmental focus of each subsystem and should be considered when interpreting age-related patterns in treatment-need categories and individual clinical findings.

The present findings are consistent with previous IPION-based studies reporting that moderate and definite preventive/interceptive orthodontic treatment need are common during mixed dentition [16,18,19]. A recent study in a Saudi Arabian population similarly reported a substantial burden of preventive and interceptive orthodontic treatment need [21], supporting the relevance of IPION-based assessment during this developmental period.

Dental caries contributed more prominently to the overall IPION profile during the early mixed dentition period. Although dental caries was common in both age groups, the number of carious teeth was significantly correlated with total IPION scores only among 6-year-old children. In addition, the number of carious teeth increased significantly across treatment-need categories in this age group, with significant differences between all pairwise comparisons. The categorical analysis similarly showed that definite treatment need was increasingly frequent from caries-free children to those with occlusal and interproximal caries. Because dental caries is itself a component of the IPION scoring system, these findings primarily demonstrate its contribution to the overall index score rather than an independent association with orthodontic treatment need.

From a biological and clinical perspective, untreated carious lesions during early mixed dentition may adversely affect normal occlusal development through loss of proximal contacts, reduction in available arch length, and an increased risk of premature loss of primary teeth. These changes may influence the eruption pathway of the permanent dentition and contribute to the development or aggravation of occlusal irregularities [22,23]. The present findings are also consistent with IPION-based studies reporting a substantial burden of dental caries among children requiring preventive or interceptive orthodontic care [19,24]. Within the context of the present analysis, however, the observed association should be interpreted as reflecting the role of caries within the multidimensional IPION framework rather than as evidence that caries independently predicts orthodontic treatment need.

In contrast, no statistically significant correlation between the number of carious teeth and total IPION score was observed in the 9-year-old group, and caries categories did not differ significantly across treatment-need categories. One possible explanation is that, at later stages of mixed dentition, the overall IPION profile incorporates a broader combination of developmental and occlusal characteristics, including sagittal and transverse discrepancies, as well as eruption disturbances and space-related conditions [25]. The relative contribution of dental caries to the overall IPION profile may therefore become less prominent as the number and diversity of other age-specific findings increase. This pattern suggests that the contribution of caries to the overall IPION score may be more pronounced during early mixed dentition, whereas the later mixed-dentition profile reflects a wider range of index components.

This broader perspective distinguishes the IPION from conventional orthodontic treatment-need indices developed primarily for the permanent dentition. The IPION integrates orthodontic, dental, and developmental findings relevant to mixed dentition [12,16,17], providing an age-specific framework that extends beyond established malocclusion alone.

Premature loss of primary teeth also contributed prominently to the overall IPION profile, particularly in the early mixed dentition period. Among 6-year-old children, the number of prematurely lost teeth showed a strong positive correlation with total IPION scores and increased significantly across treatment-need categories, with significant differences between all pairwise comparisons. Categorical analysis also demonstrated a marked difference according to premature tooth-loss status: definite treatment need was present in 77.8% of children with premature tooth loss compared with 29.1% of those without premature tooth loss. As premature tooth loss is itself included in the IPION scoring system, these findings should be interpreted as reflecting its contribution to the total IPION profile rather than an independent effect on orthodontic treatment need.

The pattern was less pronounced among 9-year-old children. The number of prematurely lost teeth showed only a weak positive correlation with total IPION score. When premature tooth loss was analyzed as a count, an overall difference was observed across the three treatment-need categories, although none of the Bonferroni-adjusted pairwise comparisons reached statistical significance. Similarly, when premature tooth loss was dichotomized as absent or present, its distribution across treatment-need categories did not reach statistical significance. The relatively small sample sizes within some treatment-need subgroups may also have limited the precision of these comparisons. Overall, these findings indicate a greater contribution of premature tooth loss to the IPION profile in early than in late mixed dentition.

Premature loss of primary teeth can influence occlusal development through mesial migration of adjacent teeth, reduction in arch length, and disturbances in the eruption pathways of permanent successors [26,27]. Systematic reviews have similarly reported associations with subsequent malocclusion, although the clinical consequences vary according to the tooth involved, timing of loss, dental age, and individual growth characteristics [23,28].

The stronger contribution observed in the younger group should therefore be understood within the structure of the IPION rather than as evidence that premature tooth loss independently predicts orthodontic treatment need. As mixed dentition progresses, however, preventive/interceptive orthodontic treatment need is increasingly determined by a wider range of developmental factors, including eruption patterns, sagittal and transverse discrepancies, and craniofacial growth. Premature tooth loss should therefore be considered one component of a broader developmental profile rather than an isolated determinant of orthodontic treatment need.

In addition to dental caries and premature tooth loss, several occlusal characteristics differed between the two age groups. Class II and Class III molar relationships, increased overjet, and greater vertical incisor overlap were more frequent among 9-year-old children. Posterior crossbite was also descriptively more frequent in the older group, although the between-group difference was not statistically significant. These findings are consistent with the dynamic nature of mixed dentition and with previous IPION-based evidence showing age-group differences in clinical findings [1,9,29].

Although the present cross-sectional study cannot determine the natural progression of individual malocclusions, these differences highlight the clinical relevance of orthodontic assessment throughout mixed dentition. Conditions such as posterior crossbite, increased overjet, and excessive vertical overlap may warrant early recognition and further orthodontic evaluation, as clinical priorities may vary with growth and dental development [5,6,9]. Thus, orthodontic screening should not necessarily be viewed as a single assessment at one chronological age but rather as part of continuing evaluation during dental development.

Taken together, the present findings support the mixed dentition period as an important window for preventive and interceptive orthodontic assessment. The differing patterns observed between 6- and 9-year-old children indicate that the IPION-defined treatment-need profile differs between early and late mixed dentition age groups and that the relative contribution of individual clinical factors may vary according to developmental stage. The stronger contribution of dental caries and premature primary tooth loss among younger children further supports integrating dental health and occlusal assessment during early mixed dentition.

These findings also emphasize the value of interdisciplinary collaboration between pediatric dentists and orthodontists. Pediatric dental examinations provide an opportunity to recognize both oral health problems and developing occlusal disturbances, while orthodontic assessment should consider dental and developmental conditions affecting the arches. Coordinated screening and referral may therefore support the early recognition and further evaluation of children with developing orthodontic concerns.

The present study has several strengths. First, preventive/interceptive orthodontic treatment need was evaluated using the age-specific IPION-6 and IPION-9 subsystems, enabling assessment of both early and late mixed dentition periods. Second, the study examined not only treatment-need categories but also individual dental and occlusal findings in relation to total IPION scores and treatment-need categories, providing a more detailed characterization of the clinical profile. Third, dental, developmental, and occlusal characteristics were evaluated using a standardized clinical examination protocol. Finally, the involvement of specialists in both pediatric dentistry and orthodontics provided an interdisciplinary framework for the clinical assessment of children during mixed dentition. Together, these features strengthen the clinical relevance of the findings and provide a broader perspective on IPION-defined preventive/interceptive orthodontic treatment need.

The findings should nevertheless be interpreted in light of some limitations. First, the cross-sectional design precludes conclusions regarding causal relationships or longitudinal changes in preventive/interceptive orthodontic treatment need. Because the study compared two independent age groups rather than following the same children from early to late mixed dentition, the observed between-group differences cannot be interpreted as within-individual developmental changes over time. Second, the study was conducted at a single university center and included children presenting for initial examination at the Pediatric Dentistry and/or Orthodontics clinics. Therefore, the observed treatment-need frequencies reflect this clinical sample and should not be interpreted as population prevalence. Third, although examinations were performed by experienced specialists according to a standardized IPION protocol and predefined assessment criteria, formal intra- and inter-examiner reliability coefficients were not calculated; therefore, examiner agreement could not be quantified, and some degree of measurement variability in clinically assessed IPION components and the resulting treatment-need classifications cannot be excluded. Fourth, because IPION-6 and IPION-9 are age-specific subsystems with different sets of clinical variables and scoring structures, raw total scores should be interpreted within the context of their respective subsystems, and the resulting treatment-need categories should be regarded as index-based screening classifications rather than clinically confirmed indications for orthodontic treatment. The relatively small sample sizes within some treatment-need subgroups, particularly in the 9-year-old group, may also reduce the precision and stability of subgroup comparisons. Finally, dental caries and premature loss of primary teeth are themselves components of the IPION scoring system; consequently, their relationships with total IPION scores and treatment-need categories should be regarded as internal descriptive associations within the index rather than as evidence of independent predictive effects.

Future multicenter studies involving larger and more diverse populations are needed to assess the generalizability of the IPION system. Prospective longitudinal studies are particularly important for determining whether early IPION profiles and specific clinical findings are associated with subsequent orthodontic treatment requirements and for clarifying the prognostic relevance of findings identified during mixed dentition.

## 5. Conclusions

IPION-defined preventive/interceptive orthodontic treatment need was common within this university-based clinical sample during both the early and late mixed dentition periods, with definite treatment need being the most frequent category in both age groups and a modest difference in treatment-need distribution between them. Dental caries and premature loss of primary teeth contributed more prominently to the overall IPION profile during the early mixed dentition period, highlighting the importance of considering dental health alongside occlusal and developmental characteristics when assessing growing children.

The age-specific IPION system provides a comprehensive framework for screening preventive/interceptive orthodontic treatment needs by integrating orthodontic, dental, and developmental findings relevant to the mixed dentition period. Its use may facilitate the early identification of children who may benefit from further orthodontic evaluation and may support interdisciplinary clinical decision-making between pediatric dentists and orthodontists. Incorporating age-specific orthodontic screening into broader pediatric oral health strategies may support earlier recognition and referral of children with developing orthodontic concerns. Whether IPION-defined treatment need identified during mixed dentition is associated with subsequent orthodontic treatment requirements or treatment complexity requires longitudinal investigation.

## Figures and Tables

**Table 1:** Clinical parameters and scoring criteria used in the IPION assessment.

Clinical Parameter	Scoring Criteria
Dental caries	0: No caries; 1: Occlusal caries; 2: Interproximal caries
Premature tooth loss (III-IV-V)	0: No tooth loss; 1: Premature tooth loss present
Permanent molar relationship	0: Class I; 2: Class II; 5: Class III
Overjet	0: 0–3 mm; 1: 3.1–5 mm; 2: 5.1–7 mm; 3: 7.1–9 mm; 4: >9 mm
Overbite	0: Coverage of ≤2/3 of the mandibular incisor crowns; 1: Coverage of >2/3 of the mandibular incisor crowns; 2: Complete coverage
Anterior open bite	0: Absent; 1: ≤1 mm; 2: 1.1–2 mm; 3: 2.1–3 mm; 4: ≥3.1 mm
Anterior crossbite	0: Absent or edge-to-edge bite; 1: Crossbite involving 1–2 teeth; 2: Crossbite involving >2 teeth
Posterior crossbite	0: Absent; 1: Edge-to-edge bite; 2: Single-tooth crossbite; 3: Crossbite involving more than one tooth; 4: Scissor bite involving more than one tooth
Lip incompetence	0: Lips closed at rest; 1: ≤4 mm separation at rest; 2: >4 mm separation at rest
Supernumerary teeth *	Score according to the number of affected teeth
Eruption disturbance/impaction of primary molars *	Score according to the number of affected teeth
Active frenulum *	0: Absent; 1: Present
Missing permanent incisors *	Score according to the number of affected teeth
Midline diastema *	0: ≤2 mm; 1: >2 mm

* Evaluated only within the IPION-9 subsystem.

**Table 2:** Total IPION scores and treatment-need categories according to age group.

Parameter	6-Year-Old Group (*n* = 100)	9-Year-Old Group (*n* = 100)	χ^2^	*p*	Effect Size
Total IPION score	15.20 ± 10.40; 14 (0–46)	19.30 ± 12.30; 17 (0–56)	—	—	—
Treatment need			6.12	0.047	Cramér’s V = 0.18
No treatment need	14 (14.0)	4 (4.0)	—	—	—
Moderate treatment need	35 (35.0)	38 (38.0)	—	—	—
Definite treatment need	51 (51.0)	58 (58.0)	—	—	—

Values for total IPION score are presented as mean ± standard deviation and median (minimum–maximum) separately for each age-specific subsystem. Categorical variables are presented as n (%). χ^2^: Pearson chi-square statistic; Cramér’s V: effect-size measure. Treatment-need categories were classified as 0–4, 5–13, and ≥14 for IPION-6 and 0–5, 6–14, and ≥15 for IPION-9. Statistical significance was set at *p* < 0.05.

**Table 3:** Distribution and comparison of clinical findings according to age group.

Clinical Finding	Category	6-Year-Old Group, *n* (%)	9-Year-Old Group, *n* (%)	χ^2^	*p*	Cramér’s V
Caries score	No caries	50 (50.0)	32 (32.0)	20.30	<0.001	0.319
	Occlusal caries	26 (26.0)	57 (57.0)			
	Interproximal caries	24 (24.0)	11 (11.0)			
Premature tooth loss	Absent	55 (55.0)	43 (43.0)	2.80	0.091	0.118
	Present	45 (45.0)	57 (57.0)			
Permanent molar relationship	Class I	60 (61.2)	44 (44.4)	6.70	0.035	0.183
	Class II	25 (25.5)	30 (30.3)			
	Class III	13 (13.3)	25 (25.3)			
Overjet	0–3 mm	82 (82.0)	59 (59.0)	16.10 *	0.001	0.284
	3.1–5 mm	16 (16.0)	26 (26.0)			
	5.1–7 mm	2 (2.0)	11 (11.0)			
	7.1–9 mm	0 (0.0)	4 (4.0)			
Overbite	≤2/3 coverage	77 (77.0)	59 (59.0)	23.40	<0.001	0.342
	>2/3 coverage	15 (15.0)	30 (30.0)			
	Complete coverage	1 (1.0)	11 (11.0)			
	Not assessable	7 (7.0)	0 (0.0)			
Anterior open bite	Absent	48 (48.0)	54 (54.0)	9.10 *	0.099	0.213
	≤1 mm	16 (16.0)	18 (18.0)			
	1.1–2 mm	14 (14.0)	12 (12.0)			
	2.1–3 mm	12 (12.0)	10 (10.0)			
	≥3.1 mm	3 (3.0)	6 (6.0)			
	Not assessable	7 (7.0)	0 (0.0)			
Anterior crossbite	Absent	75 (75.0)	64 (64.0)	2.80	0.237	0.118
	1–2 teeth	12 (12.0)	18 (18.0)			
	>2 teeth	13 (13.0)	18 (18.0)			
Posterior crossbite	Absent	52 (52.0)	36 (36.0)	8.40 *	0.078	0.205
	Edge-to-edge bite	18 (18.0)	24 (24.0)			
	Single-tooth crossbite	17 (17.0)	19 (19.0)			
	Crossbite involving >1 tooth	13 (13.0)	17 (17.0)			
	Scissor bite involving >1 tooth	0 (0.0)	4 (4.0)			
Lip incompetence	Absent	83 (83.0)	86 (86.0)	0.503 *	0.895	0.050
	≤4 mm separation	14 (14.0)	11 (11.0)			
	>4 mm separation	3 (3.0)	3 (3.0)			

*n* (%): number and percentage of children. Percentages for permanent molar relationship were calculated among assessable participants (*n* = 98 in the 6-year-old group and *n* = 99 in the 9-year-old group). * *p*-values were estimated using the Monte Carlo simulation method because expected cell frequencies did not satisfy Pearson chi-square assumptions. Cramér’s V was reported as the effect-size measure. Statistical significance was set at *p* < 0.05.

**Table 4:** Correlations of carious and prematurely lost tooth counts with total IPION scores.

Variable	6-Year-Old Group Spearman’s ρ	*p*	9-Year-Old Group Spearman’s ρ	*p*
Number of carious teeth	0.401	<0.001	0.154	0.127
Number of prematurely lost teeth	0.609	<0.001	0.216	0.031

ρ: Spearman’s rank correlation coefficient. Correlations were calculated separately within each age group. All tests were two-tailed, and statistical significance was set at *p* < 0.05. Because caries and premature tooth loss are components of the IPION scoring system, these associations should be interpreted as secondary analyses rather than independent predictive effects.

**Table 5:** Numbers of carious and prematurely lost teeth according to treatment-need category.

Age	Outcome	Need	*n*	Mean ± SD	Median (Min–Max)	H	*p*	Pairwise *p* *
6 years	Caries	No	14	0.07 ± 0.27	0 (0–1)	15.43	<0.001	No–Mod: 0.002
		Moderate	35	0.74 ± 0.89	0 (0–2)			No–Def: <0.001
		Definite	51	1.12 ± 0.91	1 (0–2)			Mod–Def: 0.031
9 years	Caries	No	4	0.50 ± 0.58	1 (0–1)	2.17	0.339	—
		Moderate	38	0.89 ± 0.76	1 (0–2)			—
		Definite	58	1.03 ± 0.79	1 (0–2)			—
6 years	Premature loss	No	14	0.07 ± 0.27	0 (0–1)	23.70	<0.001	No–Mod: 0.001
		Moderate	35	0.43 ± 0.92	0 (0–4)			No–Def: <0.001
		Definite	51	1.10 ± 1.01	1 (0–4)			Mod–Def: 0.002
9 years	Premature loss	No	4	0.00 ± 0.00	0 (0–0)	6.30	0.043	No–Mod: 0.133
		Moderate	38	0.66 ± 0.78	1 (0–4)			No–Def: 0.081
		Definite	58	0.90 ± 0.93	1 (0–4)			Mod–Def: 0.648

* Bonferroni-adjusted pairwise *p*-values. No: No treatment needed; Mod: moderate treatment need; Def: definite treatment need. SD: standard deviation; H: Kruskal–Wallis H statistic. All tests were two-tailed, and statistical significance was set at *p* < 0.05.

**Table 6:** Distribution of caries categories and premature tooth loss status according to treatment-need category.

Age Group	Clinical Variable	Category	No Need *n* (%)	Moderate Need *n* (%)	Definite Need *n* (%)	Total	χ^2^	*p*	Cramér’s V
6 years	Caries score	No caries	13 (26.0)	19 (38.0)	18 (36.0)	50	15.01 *	0.003	0.274
		Occlusal caries	1 (3.8)	9 (34.6)	16 (61.5)	26			
		Interproximal caries	0 (0.0)	7 (29.2)	17 (70.8)	24			
	Premature tooth loss	Absent	13 (23.6)	26 (47.3)	16 (29.1)	55	24.80	<0.001	0.498
		Present	1 (2.2)	9 (20.0)	35 (77.8)	45			
9 years	Caries score	No caries	2 (6.3)	13 (40.6)	17 (53.1)	32	1.50 *	0.881	0.087
		Occlusal caries	2 (3.5)	21 (36.8)	34 (59.6)	57			
		Interproximal caries	0 (0.0)	4 (36.4)	7 (63.6)	11			
	Premature tooth loss	Absent	4 (9.3)	17 (39.5)	22 (51.2)	43	5.50 *	0.055	0.235
		Present	0 (0.0)	21 (36.8)	36 (63.2)	57			

* Percentages are row percentages. *p*-values were estimated using the Monte Carlo simulation method because expected cell frequencies did not satisfy the assumptions of Pearson’s chi-square test. Cramér’s V was reported as the effect-size measure. Because dental caries and premature tooth loss are components of the IPION scoring system and contribute to treatment-need classification, these analyses should be interpreted as secondary descriptive comparisons rather than as independent associations with treatment need. Statistical significance was set at *p* < 0.05.

## Data Availability

The data supporting the findings of this study are available from the corresponding author upon reasonable request. The data are not publicly available due to privacy and ethical restrictions.

## Abbreviations

IPION	Index of Preventive and Interceptive Orthodontic Needs
DAI	Dental Aesthetic Index
IOTN	Index of Orthodontic Treatment Need
ICON	Index of Complexity, Outcome and Need References
